# An Amende Honorable

**Published:** 1891-04

**Authors:** 


### AN AMENDE HONORABLE.

For several years the Journal has published the proceedings of the Buffalo Medical and Surgical Association. At the meeting held in September, 1890, in reply to an inquiry, Dr. Tremaine explained the methods used by a specialist in treating hernia, and in his remarks is reported by the secretary to have used the term "notorious quack." Names were not used, but the subject and its relations were supposed to refer to Dr. Marcley, who employs electricity in the cure of hernia. A suit for damages was at once instituted, which was subsequently withdrawn on satisfactory assurance being given that the discussion was incorrectly reported, the speaker asserting that the offensive epithet was not used. Dr. Marcley then brought a suit against the editors of this Journal, with Dr. Frank H. Potter, associate editor, and Dr. William H. Bergtold, secretary of the association, for reporting and printing libelous matter, the complaint being subsequently amended by charging the defendants with reporting and printing a falsehood.

The facts in this case are as follows: In the absence of the managing editor, Dr. W. W. Potter, the proceedings of the association were given to Dr. Frank H. Potter, to whom the publication of the JOURNAL for October was entrusted, and by him were sent to the printer. The associate editor failed to critically inspect the proceedings, and they were printed as they came from the hands of the secretary. The "offensive epithet," above referred to, was not observed. The responsibility for the report of the discussion rests entirely with the secretary, and its publication with the associate editor. The responsibility of the editors depends solely on their ownership of the JOURNAL.

It must be clear to our readers that the editors could have entertained no malice towards the plaintiff in this action. Dr. Marcley is a member of the Erie County Medical Society in good and regular standing. He is, as a consequence of such membership, a reputable physician. He has undertaken the special treatment of hernia by methods which the profession do not generally employ and claims to have met with success. We are assured by him that his methods are open to the inspection of the profession, if they so desire. We take the broad position that whatever his methods, if they bring relief to suffering humanity and add to the means now used for the cure of disease, we have no words of opposition to bring against them.

The policy of the JOURNAL under its present editorial management is to avoid all personalities and to endeavor to advance in its publication the interests of the profession. It is not the organ of any clique, or school, or party. Its monthly issue is at the expense of much labor and returns to the editors only the reward given to faithful service. In the case under consideration the facts as stated will exonerate the editors from any and all intention to injure the plaintiff in this action. The publication is a matter of regret. An amicable settlement has been made and the suit is withdrawn.

